# The relationship between financial hardship and incident diabetic kidney disease in older US adults – a longitudinal study

**DOI:** 10.1186/s12882-021-02373-3

**Published:** 2021-05-05

**Authors:** Timothy R. Corwin, Mukoso N. Ozieh, Emma Garacci, Anna Palatnik, Leonard E. Egede

**Affiliations:** 1 Medical College of Wisconsin School of Medicine, Milwaukee, WI USA; 2 Center for Advancing Population Science, Medical College of Wisconsin, 8701 Watertown Plank Rd, Milwaukee, WI 53226 USA; 3 Department of Medicine, Division of Nephrology, Medical College of Wisconsin, 9200 W. Wisconsin Ave, Milwaukee, WI 53226 USA; 4 Division of Nephrology, Clement J. Zablocki VA Medical Center, Milwaukee, WI USA; 5 Department of Medicine, Division of General Internal Medicine, Medical College of Wisconsin, 9200 W. Wisconsin Ave, Milwaukee, WI 53226 USA; 6 Department of Obstetrics and Gynecology, Medical College of Wisconsin, 9200 W. Wisconsin Ave, Milwaukee, WI 53226 USA

**Keywords:** Diabetes, Diabetic kidney disease, Financial hardship, Longitudinal study

## Abstract

**Background:**

Financial hardship is associated with poor health, however the association of financial hardship and incident diabetic kidney disease (DKD) is unknown. This study aimed to examine the longitudinal relationship between financial hardship and incident DKD among older adults with diabetes.

**Methods:**

Analyses were conducted in 2735 adults age 50 or older with diabetes and no DKD using four waves of data (2006–2012) from the Health and Retirement Study, a national longitudinal cohort. The primary outcome was incident DKD. Financial hardship was based on three measures: 1) difficulty paying bills; 2) food insecurity; and 3) cost-related medication non-adherence using validated surveys. A dichotomous financial hardship variable (0 vs 1 or more) was constructed based on all three measures. Cox regression models were used to estimate the association between financial hardship, change in financial hardship experience and incident DKD adjusting for demographics, socioeconomic status, and comorbidities.

**Results:**

During the median follow-up period of 4.1 years, incident DKD rate was higher in individuals with versus without financial hardship (41.2 versus 27/1000 person years). After adjustment, individuals with financial hardship (HR 1.32, 95% CI 1.04–1.68) had significantly increased likelihood of developing DKD compared to individuals without financial hardship. Persistent financial hardship (adjusted HR 1.52 95% CI 1.06–2.18) and negative financial hardship (adjusted HR 1.54 95% CI 1.02–2.33) were associated with incident DKD compared with no financial hardship experience. However, positive financial hardship was not statistically significant in unadjusted and adjusted (adjusted HR 0.89 95% CI 0.55–1.46) models. Cost-related medication non-adherence (adjusted HR 1.43 95% CI 1.07–1.93) was associated with incident DKD independent of other financial hardship measures.

**Conclusions:**

Financial hardship experience is associated with a higher likelihood of incident DKD in older adults with diabetes. Future studies investigating factors that explain the relationship between financial hardship and incident DKD are needed.

**Supplementary Information:**

The online version contains supplementary material available at 10.1186/s12882-021-02373-3.

## Introduction

Diabetes affects about 30.3 million people in the United States [1] and about 451 million people worldwide [2]. The rise in diabetes since the 1980s has corresponded with rises in diabetic kidney disease (DKD) [3], a common long-term complication affecting about 40% of individuals with diabetes [4]. Individuals with DKD are at increased risk of cardiovascular disease [4] and progressing to end-stage renal disease [5]. DKD is also associated with significantly increased cost [6] and mortality risk [7]. While the role of risk factors such as hyperglycemia [8] and high blood pressure [9] in DKD incidence are established, growing evidence supports the role of social determinants in diabetes outcomes [10].

Social determinants of health are the conditions in which individuals are born, grow, live, work, and age [11]. These conditions exacerbate health disparities at global, national, local, and individual levels, while also impacting individuals’ socioeconomic status [12]. The established metrics of socioeconomic status are education, occupation, and income [13], however these have been shown to often be insufficient in capturing the true burden experienced by individuals [14]. Financial hardship is a measure that accounts for material conditions [15], psychological response [16, 17], and coping behaviors [18] related to interaction with the healthcare system [19, 20]. It considers the lack of available funds or resources with downstream effects [21], providing a more thorough depiction of experiences tied to socioeconomic status.

Financial hardship has been shown to have significant associations with health [22]. In particular, it has been identified as a possible risk factor for poor cardiovascular health, recurrence of coronary artery disease events [23–25] and death [22]. The exact mechanism by which financial hardship impacts health outcomes may be related to non-biologic (e.g. access to resources) and/or biologic (e.g. stress, inflammation) factors [26, 27]. For example, a longitudinal cohort study of African Americans examined the impact of financial hardship on incident coronary heart disease and found that individuals with moderate to high stress were more likely to have incident coronary heart disease although this relationship was explained by risk factors such as diabetes [23].

Prior research studies have examined the relationship between financial hardship and health outcomes in individuals with diabetes [28, 29]. However, studies investigating the association of financial hardship and complications of diabetes are sparse. To address this knowledge gap, we first examined the longitudinal relationship between financial hardship and incident DKD amongst older US adults with diabetes, and examined the longitudinal relationship between incident DKD and change in financial hardship experience. Our first hypothesis was that there is a positive longitudinal relationship between financial hardship and incident DKD amongst older US adults with diabetes. The second hypothesis was that “positive”, “negative” and “persistent” financial hardship change is positively associated with incident DKD amongst older US adults with diabetes.

## Methods

### Study design and population

The Health and Retirement Study (HRS) is a longitudinal panel study that surveys a representative sample of approximately 20,000 people every 2 years in America, supported by the National Institute on Aging and the Social Security Administration [30]. The HRS interviews focuses on the health, economics, and demographics of aging and the retirement process. Since 2006, a Leave-Behind Questionnaire on psychosocial topics has been utilized to obtain information about participants’ evaluation of their life circumstances, subjective well-being, and lifestyle. This psychosocial information is obtained in each biennial wave from a rotating (random) 50% of the core panel participants who complete the enhanced face-to-face interview [31]. Longitudinal data are available at four-year intervals.

This study cohort included 4 core interviews from 2006 to 2012. There were 6623 participants aged 50 years and older with self-reported diagnosis of diabetes and defintive answer (yes/no) to the question “kidney trouble due to diabetes” eligible for inclusion, see supplement Figure 1. Among them, 4442 participants completed financial hardship-related information and 3971 participants reported ‘no’ to the question “kidney trouble due to diabetes” in the first available financial hardship related information interview. We defined our cohort as the 2735 participants who reported ‘no’ to the question “kidney trouble due to diabetes”, completed all the financial hardship related information and had at least one follow-up interview during the study period. The cohort baseline was defined as participants first available interview with complete financial hardship-related information *plus* a ‘no’ response to the question “kidney trouble due to diabetes”. Participants could have a maximum of four interviews during the study period. For participants with more than one follow-up interview, we selected the last interview as the end of follow-up and DKD event was defined as the first interview post-baseline with ‘yes’ response to the question “kidney trouble due to diabetes”. Participants without a ‘yes’ response were censored at the last interview with a ‘no’ response to the question “kidney trouble due to diabetes”.

We divided the cohort into two groups based on the presence or absence of financial hardship. Of the 2735 participants (group 1), 1535 participants who had financial hardship information reported during the follow-up interview were used for change in financial hardship experience analysis (group 2). See supplement Figure 1.

### Primary outcome

The primary outcome for this study was incident DKD among adults with self-reported diabetes, based on ‘yes’ to the question: “has a doctor ever told you that you have diabetes or high blood sugar?”. DKD was based on the question: “has your diabetes caused you to have trouble with your kidneys or protein in your urine?”

The start date (baseline) was defined as the date of the first interview during the study period (2006–2012). The event (incident DKD) date was defined as the date study participants first reported ‘yes’ to the question “kidney trouble due to diabetes” during follow-up interviews. The event date was censored on the last interview date if no DKD was reported. HRS recorded year and month of interviews. We used the 15th day of the month to construct the start date, event date and censor date. Length of follow-up was calculated from the start date to either the event date or censor date. The follow-up time was presented in years with an average person-years of follow-up of 10,686.

### Independent variable

#### Financial hardship

Financial hardship was the primary predictor for this study. Financial hardship was constructed using three measures: 1) difficulty paying bills; 2) food insecurity; and 3) cost-related medication non-adherence based on validated surveys from the biennial core interview and RAND data sets [31].

1. Difficulty paying bills was based on the question “how difficult is it for you/your family to meet monthly payments on your/your family’s bills?” and categorized as “yes” (somewhat/very/completely difficult) vs. “no” (not at all/no very difficult) depending on participants’ response.
2. Food insecurity was categorized as “yes” when participants answered “yes” to the question “In the last 12 months, did you ever eat less than you felt you should because there wasn’t enough money to buy food?”, categorized as “no” when answered “no” to the above quesiton, or “yes” to question “In the last two years, have you always had enough money to buy the food you need?”.
3. Cost-related medication non-adherence was categorized as “yes” vs. “no” based on the question “have you ended up taking less medication than was prescribed for you because of the cost?”.

A dichotomous financial hardship variable (0 vs ≥1) was generated by coding all “yes” responses as 1 and “no” responses as 0. Financial hardship was defined as a score of ≥1. Additionally, a 4-category change in financial hardship experience variable was created for individuals who answered the financial hardship question during the follow-up interview as follows: a) Individuals who reported no financial hardship in both interviews were categorized as “No Financial Hardship”; b) Individuals who reported financial hardship in both interviews were categorized as “Persistent Financial Hardship”; c) Individuals who reported financial hardship in the first interview and reported no financial hardship in the second interview were categorized as “Positive Financial Hardship Change”; d) Individuals who reported no financial hardship in the first interview and reported financial hardship in the second interview were categorized as “Negative Financial Hardship Change”.

### Demographic factors and covariates

Demographic factors included gender, age (in years), race/ethnicity (categorized as non-Hispanic White; non-Hispanic Black; Hispanic; and other minority), marital status (categorized as yes or no based on response to “married or living with a partner”). Socioeconomic factors included education (categorized as no degree, high school diploma/GED, and higher education), household income and assets (grouped into four quarters for all participants). Comorbidities were included as a count variable based on the total sum (maximum of 7) of the following chronic diseases reported: high blood pressure, cancer, lung disease, heart condition, stroke, emotional/psychiatric problems, arthritis. Duration of diabetes was calculated from self-reported “year diabetes first diagnosed” to interview time in years. All data came from the biennial core interview data, except total wealth and household income which came from RAND data.

### Statistical analysis

Means or proportions of the baseline characteristics were calculated for the entire cohort, participants without DKD and financial hardship (reference group) and participants without DKD and with financial hardship. Incident DKD rates per 1000 person-years of follow-up was calculated for the two groups. Time to event for the two groups were estimated using Kaplan-Meier method.

Hierarchical Cox proportional hazards regression models were used to investigate the longitudinal relationship between financial hardship and incident DKD compared with the reference group. First, univariate Cox models were used to examine the relationship between financial hardship as the primary predictor variable and incident DKD as the outcome. Then we ran four multivariable Cox models by adding the following variable groups in sequence: a) demographics (age, gender, race/ethnicity, marital status), b) socioeconomic (education, household income and assets); c) comorbidity count, d) duration of diabetes. We further investigated whether change in financial hardship was associated with patient reported incident DKD. Finally, the three individual financial hardship measures (difficulty paying bills, food insecurity, and cost-related medication non-adherence) were entered together to investigate the independent effect of each on incident DKD. Sensitivity analyses were performed by moving the start date forward by 12 months and results remained consistent.

All *p*-values were 2-sided and *p* < 0.05 was considered statistically significant. Statistical analysis was performed with SAS version 9.4 (SAS Institute).

## Results

### Characteristics at baseline

The average duration of follow-up for this longitudinal cohort of 2735 adults was approximately 4 years (10,686 person years of follow-up). A total of 347 (13%) adults developed incident DKD. Table 1 shows the baseline characteristics of the study participants by absence or presence of financial hardship. The mean age in this cohort was 68 ± 9.2 years and adults with financial hardship had a mean age of 65 ± 8.8 years. Compared with adults without financial hardship, adults with financial hardship were more likely to be female, ethnic minority, less educated and to have a low household income.

**Table 1:** Baseline characteristics for older adults with diabetic kidney disease by absence or presence financial hardship

	All	Without Financial Hardship	With Financial Hardship	*p*-value
	*n* = 2735	*n* = 1648	*n* = 1087	< 0.01
**Age (years)**				
Mean ± ^a^SD	68.1 ± 9.2	69.8 ± 9.0	65.4 ± 8.8	
**Gender**				< 0.01
Female	55.17%	49.09%	64.40%	
**Race/Ethnicity**				< 0.01
NH White	65.27%	72.94%	53.63%	
NH Black	19.05%	14.14%	26.49%	
Hispanic	12.87%	10.19%	16.93%	
Other	2.82%	2.73%	2.94%	
**Married or living with a partner**				< 0.01
Yes	66.14%	71.06%	58.69%	
**Education level**				< 0.01
No degree	24.50%	20.08%	31.19%	
High school diploma/GED	53.97%	54.73%	52.81%	
Higher education	21.54%	25.18%	16.01%	
**Household income and assets**				< 0.01
1st Quartile	26.69%	15.47%	43.70%	
2nd Quartile	27.71%	24.76%	32.20%	
3rd Quartile	25.70%	31.55%	16.84%	
4th Quartile	19.89%	28.22%	7.27%	
^b^**Comorbidity count**				< 0.01
Mean ± ^a^SD	2.3 ± 1.3	2.2 ± 1.2	2.4 ± 1.3	
**Duration of diabetes (year)**				
Mean ± ^a^SD	9.0 ± 8.7	8.9 ± 8.9	9.0 ± 8.4	0.74

^a^*SD* standard deviation; ^b^Comorbidity count includes the following comorbidities: high blood pressure, cancer, lung disease, heart condition, stroke, emotional/psychiatric problems, arthritis

### Longitudinal relationship between financial hardship and patient reported incident DKD

**Fig. 1:**
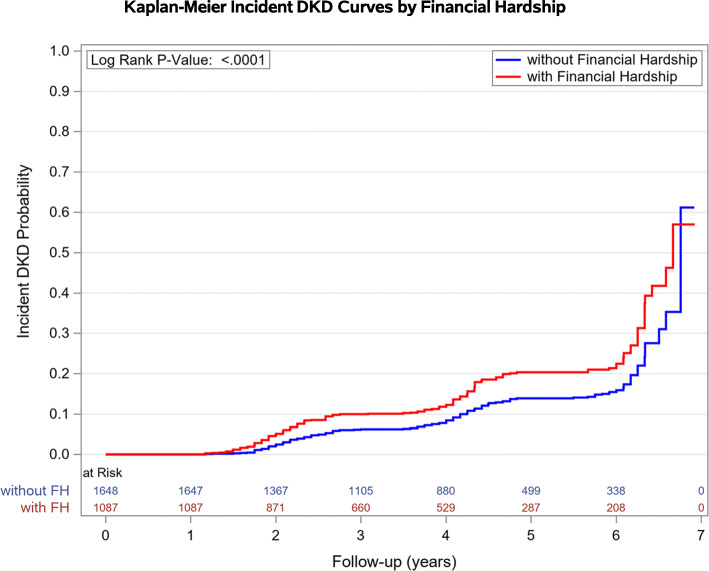
Estimated probability of incident diabetic kidney disease (DKD) according to financial hardship experience

Incident DKD rate per 1000 person years of follow-up was higher in individuals with financial hardship (41.2 per 1000 person years) compared with individuals without financial hardship experience (27 per 1000 person years), see Fig. 1 and Table 2. Univariate (unadjusted) and multivariate-adjusted HRs of incident DKD was higher for people with financial hardship compared with people without financial hardship. In the unadjusted model, individuals reporting financial hardship were 54% more likely to develop DKD compared with individuals with no financial hardship (HR 1.54 95% CI 1.25–1.91) during the follow-up period. After adjusting for demograhics (model 1), the relationship between financial hardship experience and incident DKD remained significant (HR 1.59 95% CI 1.27–1.99). Statistical significance was maintained in model 4 adjusting for demographics, socioeconomic status, comorbidity count, and duration of diabetes (HR 1.32 95% CI 1.04–1.68).

**Table 2:** Univariate and multivariate-adjusted hazard ratio of incident diabetic kidney disease according to financial hardship experience

	Without Financial Hardship	With Financial Hardship	p-value
	n = 1648	n = 1087	
Person-years of follow-up	6581	4105	
DKD (n)	178	169	
DKD rate (per 1000)	27.0	41.2	
Hazard Ratio (95% CI)			
Univariate model	1.00 (ref)	1.54 (1.25–1.91)	< 0.01
Multivariate model 1	1.00 (ref)	1.59 (1.27–1.99)	< 0.01
Multivariate model 2	1.00 (ref)	1.42 (1.12–1.79)	< 0.01
Multivariate model 3	1.00 (ref)	1.30 (1.03–1.65)	0.03
Multivariate model 4	1.00 (ref)	1.32 (1.04–1.68)	0.02

Multivariate Model 1: adjusted by demographics (age, gender, race/ethnicity, marital status);

Multivariate Model 2: adjusted by demographics and socioeconomic status (education, household income and assets);

Multivariate Model 3: adjusted by demographics, socioeconomic status, and comorbidity count

Multivariate Model 4: adjusted by demographics, socioeconomic status, comorbidity count, and duration of diabetes

### Longitudinal relationship between change in financial hardship experience and patient reported incident DKD

Incident DKD was highest in people with persistent financial hardship (51 per 1000 person years) and lowest in people with positive financial hardship change (30 per 1000 person years), see Table 3. Univariate and multivariate-adjusted HRs of incident DKD were significantly higher for people with negative (ie. transition from no financial hardship experience into financial hardship) and persistent financial hardship compared with people experiencing positive financial hardship change (ie. transition from experiencing financial hardship into no financial hardship) or no financial hardship. In the unadjusted model, negative financial hardship change (HR 1.66 95% CI 1.11–2.47) and persistent financial hardship (HR 1.78 95% CI 1.30–2.44) were significantly associated with incident DKD relative to those with no financial hardship. Positive financial hardship change was not statistically significant (HR 1.06 95% CI 0.66–1.69). After adjustments, negative financial hardship change (HR 1.54 95% CI 1.02–2.33) and persistent financial hardship (HR 1.52 95% CI 1.06–2.18) maintained statistical significance. A positive change in financial hardship experience remained not statistically significant (HR 0.89 95% CI 0.55–1.46).

**Table 3:** Univariate and multivariate-adjusted hazard ratio of incident diabetic kidney disease according to change in financial hardship experience

	No Financial Hardship	Persistent Financial Hardship	Positive Financial Hardship Change	Negative Financial Hardship Change	p-value
	*n* = 757	*n* = 389	*n* = 186	*n* = 203	
Person-years of follow-up	3120	1.568	759	820	
DKD count	79	80	23	35	
DKD rate (per 1000)	25.3	51.0	30.3	42.7	
Hazard Ratio (95% CI)					
Univariate model	1.00 (ref)	1.78 (1.30–2.44)	1.06 (0.66–1.69)	1.66 (1.11–2.47)	< 0.01
Multivariate model 1	1.00 (ref)	1.66 (1.20–2.31)	0.99 (0.62–1.59)	1.58 (1.06–2.37)	< 0.01
Multivariate model 2	1.00 (ref)	1.55 (1.09–2.21)	0.95 (0.59–1.54)	1.57 (1.04–2.36)	0.02
Multivariate model 3	1.00 (ref)	1.47 (1.03–2.11)	0.87 (0.53–1.42)	1.50 (1.00–2.27)	0.03
Multivariate model 4	1.00 (ref)	1.52 (1.06–2.18)	0.89 (0.55–1.46)	1.54 (1.02–2.33)	0.02

Multivariate Model 1: adjusted by demographics (age, gender, race/ethnicity, marital status)**;**

Multivariate Model 2: adjusted by demographics and socioeconomic status (education, household income and assets)**;**

Multivariate Model 3: adjusted by demographics, socioeconomic status, and comorbidity count

Multivariate Model 4: adjusted by demographics, socioeconomic status, comorbidity count, and duration of diabetes

### Independent effect of individual financial hardship measures and patient reported incident DKD

Incident DKD was highest in individuals reporting food insecurity (51 per 1000 person years) followed by individuals reporting cost-related medication non-adherence (49 per 1000 person years) and lowest in individuals reporting difficulty paying bills (40 per 1000 person years), see Table 4. In the unadjusted model, difficulty paying bills (HR 1.45 95% CI 1.17–1.80), cost-related medication non-adherence (HR 1.69 95% CI 1.29–2.22), and food insecurity (HR 1.73 95% CI 1.10–2.72) were all significantly associated with incident DKD. In the fully adjusted model, cost-related medication non-adherence maintained significance (HR 1.43 95% CI 1.07–1.93), while difficulty paying bills (HR 1.15 95% CI 0.90–1.47) and food insecurity (HR 1.01 95% CI 0.62–1.66) did not.

**Table 4:** Independent effect of financial hardship measures on incident diabetic kidney disease

	^a^Difficulty paying bills	p-value	^a^Cost-related medication non-adherence	p-value	^a^Food insecurity	p-value
	*n* = 952		*n* = 358		*n* = 122	
Person-years follow-up	3613		1299		395	
DKD count	146		64		20	
DKD rate (per 1000)	40.4		49.3		50.7	
Hazard Ratio (95% CI)						
Univariate model	1.45 (1.17–1.80)	< 0.01	1.69 (1.29–2.22)	< 0.01	1.73 (1.10–2.72)	0.02
Multivariate model 1a	1.32 (1.05–1.65)	0.02	1.48 (1.10–1.98)	< 0.01	1.27 (0.79–2.04)	0.33
Multivariate model 1b	1.36 (1.07–1.72)	0.01	1.52 (1.13–2.03)	< 0.01	1.36 (0.84–2.20)	0.22
Multivariate model 2	1.22 (0.96–1.56)	0.11	1.45 (1.08–1.95)	0.01	1.21 (0.74–1.97)	0.44
Multivariate model 3	1.16 (0.90–1.48)	0.25	1.40 (1.05–1.88)	0.02	1.03 (0.63–1.68)	0.92
Multivariate model 4	1.15 (0.90–1.47)	0.26	1.43 (1.07–1.93)	0.02	1.01 (0.62–1.66)	0.96

^a^Model reference groups: Absence of financial hardship measures (difficulty paying bills, cost-related medication non-adherence and food security)

Multivariate Model 1a: model included all three financial hardship measures - difficulty paying bills, cost-related medication non-adherence and food insecurity;

Multivariate Model 1b: all three financial hardship measures adjusted by demographics (age, gender, race/ethnicity, marital status)**;**

Multivariate Model 2: all three financial hardship measures adjusted by demographics and socioeconomic status (education, household income and assets)**;**

Multivariate Model 3: all three financial hardship measures adjusted by demographics, socioeconomic status, and comorbidity count

Multivariate Model 4: all three financial hardship measures adjusted by demographics, socioeconomic status, comorbidity count, and duration of diabetes

## Discussion

In this longitudinal cohort of older US adults with diabetes, we found that individuals reporting financial hardship experience were significantly more likely to develop DKD compared to those who did not report financial hardship experience after adjusting for clinically relevant covariates. In addition, individuals who report persistent financial hardship were significantly more likely to develop DKD compared with those reporting no financial hardship experience. This study also suggests that cost-related medication non-adherence is associated with patient reported incident DKD independent of other financial hardship measures, demographic factors, socioeconomic status and comorbidity burden.

To our knowledge this is the first longitudinal study to examine the impact of financial hardship experience on diabetes complication. We found that individuals who report financial hardship experience had a 30% increased risk of incident DKD compared with individuals who did not report financial hardship. These results suggest that financial hardship experience, particularly persistent financial hardship is detrimental to diabetes outcomes. Our study findings are consistent with prior research studies which suggest financial hardship experience has an inverse relationship with health and health outcomes [22–26, 29, 32]. Tucker-Seely et al. found that even after controlling for demographics, socioeconomic status and functional limitations, the number and types of financial hardships was associated with a higher probability of death in older Americans [22]. Similarly, a cross-sectional study showed that financial strain was associated with lower ideal cardiovascular health, and found that the effect of financial strain on cardiovascular health is cumulative, with increasing number of financial strain events being associated with worse cardiovascular health [24]. The study defined financial strain using acute life events such as unemployment, moving to a worse residence or neighborhood, serious financial problems, difficulty paying bills and perception of financial situation, which differs from our study.

This study uniquely examines the longitudinal relationship of change in financial hardship experience and patient reported incident DKD. Our results suggest that individuals who experience persistent and negative financial hardship change (ie. transition from no financial hardship into experience of financial hardship) may be at a higher risk of incident DKD. In the model adjusting for demographic, socioeconomic status, comorbidity and duration of diabetes, persistent and negative financial hardship experience were associated with 52 and 54% increased risk of patient reported incident DKD respectively. Positive financial hardship change (ie. transition from reporting financial hardship into no financial hardship experience) had no association with patient reported incident DKD. The underlying mechanism for this relationship is unclear. It is plausible that the detrimental impact of financial hardship experience with regards to incident DKD is irreversible. A standard measure for change in financial hardship experience that captures duration of experience is necessary for further investigation. Meanwhile, glycemic control is established as a major determinant of DKD and existing studies show that material insecurities such as food insecurity and cost-related medication are adversely associated with diabetes control [28]. Evidence also suggests that cumulative exposure to economic hardship across adult life is associated with elevated inflammatory markers [27], which are associated with prevalent and incident chronic kidney disease [32, 33]. This may explain the association of financial hardship and patient reported incident DKD observed in our study population.

Our study results have a few significant clinical and research implications. First, this study highlights the need to identify and address social risk factors such as financial hardship among older adults, in addition to managing the traditional risk factors for diabetes complications. It underscores the need for a multidisciplinary team of health care professionals including social workers in the care of patients with diabetes complicated by chronic kidney disease [34]. While addressing and eliminating social risk factors require policy reforms and targeted programs, health care providers may facilitate timely access to resources by direct referral or by taking advantage of social worker services when available. In addition, health care providers can incorporate social risk information into health care delivery and their medical decision making process such as medication prescription formula or dose frequency in order to improve patient outcome. This study’s findings suggest that independent of financial measures such as food insecurity and difficulty paying bills, cost-related medication non-adherence is associated with patient reported incident DKD. This is of particular importance given the public health burden of poor medication adherence [35]. Most study interventions focused on improving medication adherence in older US adults are behavioral/educational and pharmacist-led [36]. Future intervention studies targeting cost-related medication non-adherence in older adults with diabetes who experience financial hardship are needed to better understand our study findings.

Despite the strengths of this study, it has some limitations. First, substantial heterogeneity exists in the literature for measures of financial hardship which limits our ability to compare our findings with prior studies. Though the few studies that exist have included one or more of the measures of financial hardship used here, a standardized measure for financial hardship is needed. Second, diabetes and DKD was based on self-report, which likely underestimates the prevalence of DKD. According to the Centers for Disease Control and Prevention, 9 in 10 adults with chronic kidney disease are not aware that they have the disease and less than 15% of adults with moderate chronic kidney disease are aware they have the disease [37]. In addition, non-specialist health provider recognition of chronic kidney disease was 56–70%. However, self-reported history of chronic diseases such as diabetes and kidney disease that require continuing treatment have been shown to be reliable [38, 39]. Third, while we controlled for relevant covariates, the possibility of residual confounders which may independently influence the relationship between financial hardship and incident DKD amongst adults with diabetes remains. Fourth, HRS surveys an older population, which limits our ability to generalize our findings to younger population of adults with diabetes.

## Conclusions

In conclusion, in a longitudinal cohort of older US adults we show that compared with individuals without financial hardship, individuals with financial hardship experience were significantly more likely to develop DKD. In particular, individuals reporting persistent financial hardship had a higher probability of patient reported incident DKD while individuals reporting negative financial hardship change may be at risk of incident DKD. Our study findings suggest cost-related medication non-adherence may be an important target for future intervention studies to mitigate adverse outcomes. Future studies are needed to explore factors not included in this study that may explain the relationship between financial hardship and incident DKD.

## Supplementary Information

**Additional file 1: Supplement Figure 1.** Study Flow Diagram.

## Data Availability

The datasets analyzed for the current study are available at the Institute for Social Research, https://hrsdata.isr.umich.edu/data-products?_ga=2.163177980.1945844060.1613501577-348601380.1611285344

## Abbreviations

DKD: Diabetic Kidney Disease
HR: Hazard Ratio
CI: Confidence Interval
HRS: Health and Retirement Study
RAND: Research and Development
SAS: Statistical Analysis System
SD: Standard Deviation
