# Supplementary material for: The relationship between financial hardship and incident diabetic kidney disease in older US adults – a longitudinal study
Source: BMC Nephrol. 2021 May 5;22:167. doi: 10.1186/s12882-021-02373-3 (PMC8101204; doi:10.1186/s12882-021-02373-3)
Supplement: Supplementary file 1 — Additional file 1: Supplement Figure 1. Study Flow Diagram. [file 12882_2021_2373_MOESM1_ESM.docx]

## Follow-Up

## Baseline

Group 2 – Participants who responded during last available interview (end of follow-up) and responded to all financial hardship-related information *plus* first ‘yes’ to DKD question as DKD event (n=1535)

Excluded participants who responded ‘Yes’ to question “kidney trouble due to diabetes” before or at baseline interview (n=75)

Excluded participants without at least one follow-up interview (n=1161)

Group 1 – Participants who responded during last available interview (end of follow-up) *plus* first ‘yes’ to DKD question as DKD event (n=2735)

Participants with at least one follow-up interview (n=2810)

Participants who responded ‘No’ to DKD question at first available interview *plus* completed all financial hardship-related information (n=3971)

Participants who completed all financial hardship-related information (n=4442)

Participants who responded to diabetic kidney disease (DKD) ‘yes/no’ question – “*kidney trouble due to diabetes*” (n=6623)

Participants age 50 and above with self-reported diabetes during

2006-2012 (n=6804)
